# First Record of Black Band Disease in the Hawaiian Archipelago: Response, Outbreak Status, Virulence, and a Method of Treatment

**DOI:** 10.1371/journal.pone.0120853

**Published:** 2015-03-16

**Authors:** Greta S. Aeby, Thierry M. Work, Christina M. Runyon, Amanda Shore-Maggio, Blake Ushijima, Patrick Videau, Silvia Beurmann, Sean M. Callahan

**Affiliations:** 1 Hawai‘i Institute of Marine Biology, Kāne‘ohe, Hawaii, United States of America; 2 U.S. Geological Survey, National Wildlife Health Center, Honolulu Field Station, Honolulu, Hawaii, United States of America; 3 Marine Biology Graduate Program, University of Hawai‘i, Honolulu, Hawaii, United States of America; 4 Microbiology Department, University of Hawai‘i, Honolulu, Hawaii, United States of America; King Abdullah University of Science and Technology, SAUDI ARABIA

## Abstract

A high number of coral colonies, *Montipora* spp., with progressive tissue loss were reported from the north shore of Kaua‘i by a member of the Eyes of the Reef volunteer reporting network. The disease has a distinct lesion (semi-circular pattern of tissue loss with an adjacent dark band) that was first observed in Hanalei Bay, Kaua‘i in 2004. The disease, initially termed *Montipora* banded tissue loss, appeared grossly similar to black band disease (BBD), which affects corals worldwide. Following the initial report, a rapid response was initiated as outlined in Hawai‘i’s rapid response contingency plan to determine outbreak status and investigate the disease. Our study identified the three dominant bacterial constituents indicative of BBD (filamentous cyanobacteria, sulfate-reducing bacteria, sulfide-oxidizing bacteria) in coral disease lesions from Kaua‘i, which provided the first evidence of BBD in the Hawaiian archipelago. A rapid survey at the alleged outbreak site found disease to affect 6-7% of the montiporids, which is higher than a prior prevalence of less than 1% measured on Kaua‘i in 2004, indicative of an epizootic. Tagged colonies with BBD had an average rate of tissue loss of 5.7 cm2/day over a two-month period. Treatment of diseased colonies with a double band of marine epoxy, mixed with chlorine powder, effectively reduced colony mortality. Within two months, treated colonies lost an average of 30% less tissue compared to untreated controls.

## Introduction

In the Caribbean, declines in live coral began in the late 1970s when disease outbreaks that affected the major reef-forming corals, *Acropora palmata* and *A*. *cervicornis*, were first reported [1]. Since that time, coral cover has declined precipitously from an average of 50% in the 1970s to an average of 10% in 2002 [2] and coral diseases have contributed significantly to that decline [3]. Coral diseases are now degrading reefs of the Indo-Pacific [4–6]; numerous disease outbreaks have been reported from Australia [7, 8], Philippines [9], Marshall Islands and Palau [10], and even the remote reefs of Palmyra Atoll [11]. The reefs of the Indo-Pacific appear to be starting down the same pathway of disease-induced destruction experienced by the reefs in the Caribbean and Florida Keys.

Within Hawai‘i, baseline information on coral diseases has been established; disease is widespread on reefs but occurs at a low prevalence [5, 12, 13]. However, disease outbreaks are starting to occur with increasing frequency. In 2003, an outbreak of *Acropora* white syndrome caused massive mortality of the table corals (*Acropora cytherea*) at French Frigate Shoals in the northwestern Hawaiian Islands (Papahānaumokuākea Marine National Monument) [14, 15]. Additionally, outbreaks of *Montipora* white syndrome occurred in Kāne‘ohe Bay, O‘ahu in 2006 [16] and ‘Āhihi-Kīna‘u, Maui in 2008 [17]. Since coral diseases are an emerging issue in Hawai‘i, the State developed a rapid response contingency plan (RRCP) to give managers the capacity to respond to bleaching or disease events in a timely and efficient manner [18]. Protocols were established to investigate bleaching or disease events and aid in determining the significance, epizootiology, and potential causal linkages of outbreaks. A citizen science program, the Eyes of the Reef Network (http://eorhawaii.org/), was also developed to train community members in identifying and alerting managers to the occurrence of unusual disease events on Hawai‘i’s coral reefs.

**Fig 1:**
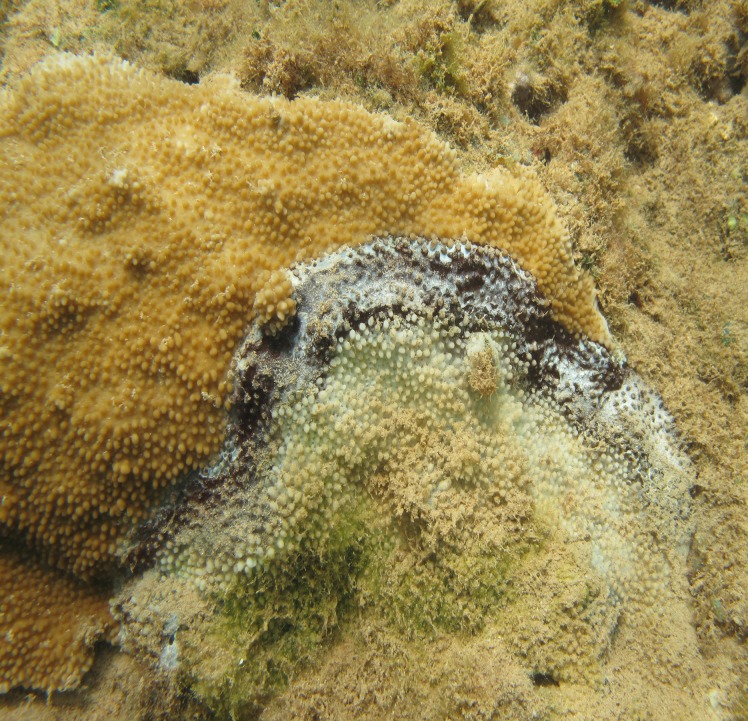
*Montipora capitata* colony with signs of black band disease.

In the summer of 2012, a member of the Eyes of the Reef (EOR) network reported a high number of coral colonies, *Montipora* spp. (*M*. *capitata*, *M*. *patula*, *M*. *flabellata*), on the north shore of Kaua‘i with progressive tissue loss. The corals had distinct disease lesions (semi-circular pattern of tissue loss with an adjacent dark band) (Fig. 1) that were first observed in Hanalei Bay, Kaua‘i in 2004 [19]. The lesion, initially termed *Montipora* banded tissue loss, appeared grossly similar to black band disease (BBD), which affects numerous coral genera worldwide [3]. BBD is caused by a microbial consortium, visually dominated by filamentous cyanobacteria, that creates the characteristic black band [20]. Other constituents of the BBD lesion include sulfide-oxidizing bacteria (*Beggiatoa* spp.), sulfate-reducing bacteria that include members of the *Desulfovibrio* genus, and numerous heterotrophic bacteria [21–23]. The cyanobacteria and sulfide-oxidizing bacteria exhibit vertical migration within the microbial mat under changing diel light conditions, which results in dynamic vertical microgradients in oxygen and sulfide [24, 25]. The sulfate-reducing bacteria are responsible for the highly concentrated sulfide and anoxic conditions underneath the BBD mat that is lethal to coral tissue [25, 26]. Following the initial report by an EOR member, a rapid response was initiated, as outlined in Hawai‘i’s RRCP, to determine outbreak status and collect data for follow-up studies. The objectives of this study were to 1) conduct rapid surveys to determine outbreak status of the disease, 2) determine disease virulence by measuring rate of tissue loss on marked colonies, 3) test lesion occlusion as a method of disease treatment, and 4) determine whether the dominant microbial constituents of the disease lesion were consistent with BBD reported from other regions of the world. Our results indicate the outbreak status of the first report of BBD in the Hawaiian archipelago and show a successful method arresting progression of lesions. The ability to identify and respond quickly to this disease outbreak was facilitated by the prior development of programs such as the Eyes of the Reef network and Hawai‘i’s Rapid Response Contingency Plan.

## Methods

### Ethics statement

This research was conducted under Special Activity Permit No. 2013–16 issued by the Hawai‘i Division of Aquatic Resources.

### Rapid surveys

To determine disease prevalence (number of infected colonies/total colonies examined) at Anini, which is the reported outbreak site (22.227°N, 159.456°W), a diver swam from shore out to the reef crest and all *Montipora* spp. colonies along an approximately 6 meter wide swath were examined and enumerated. All diseased colonies were photographed and enumerated. Surveys were conducted in October 2012.

### Disease virulence and lesion occlusion for disease treatment

**Fig 2:**
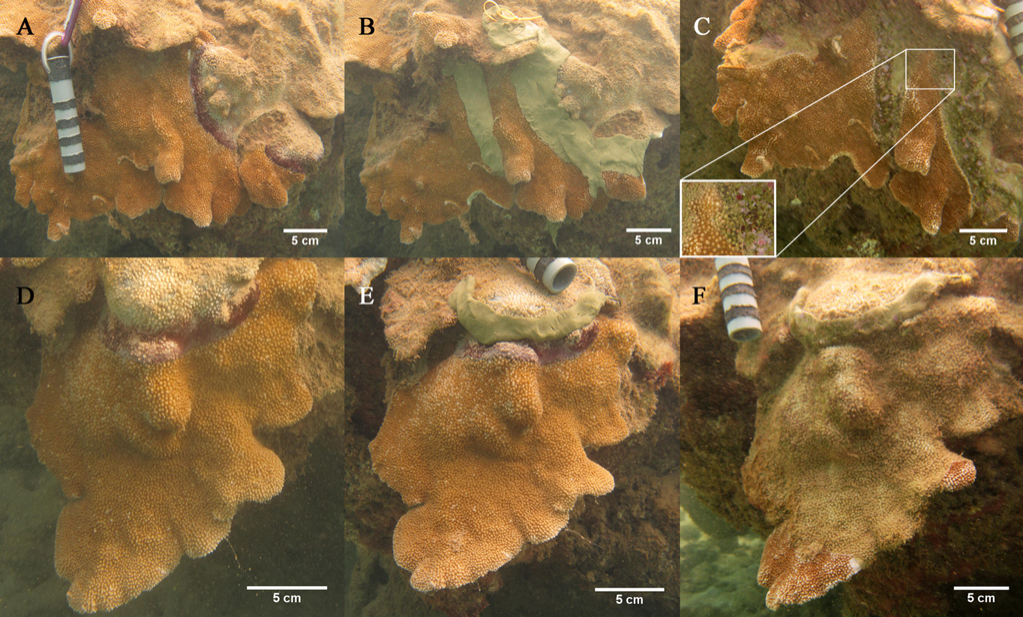
The lesion occlusion method of disease treatment. A) Infected *M*. *capitata* before treatment. B) Colony with marine epoxy over lesion and with a band of epoxy placed as a “firebreak” approximately 5 cm away. C) Same colony two months post-treatment. Note that the marine epoxy is overgrown with algae and the edge of the lesion is starting to grow over the epoxy. D) Control infected *M*. *capitata* before marking with marine epoxy. E) Colony with marine epoxy approximately 5 cm behind lesion. F) Control colony after two months.

The rate of tissue loss on diseased colonies (n = 8) and colonies treated using the lesion occlusion method (n = 8) was measured by tagging and photographing colonies (*M*. *capitata*) though time. To measure disease progression, a band of marine epoxy (ZSPAR Splash Zone) was applied to the eight control colonies on bare skeleton roughly two centimeters behind the leading edge of the lesion. For the eight treatment colonies, the bacterial mat associated with disease lesions was loosened from the coral surface using the flat edge of a dive knife and removed by suction with a sterile 50cc syringe and used for identification of select bacteria as described below. To increase the probability of killing any potential bacterial pathogens, the marine epoxy was mixed with chlorine powder (calcium hypochlorite) (~15mL/ 50 mL epoxy), and then spread over the border of live tissue and bare skeleton (primary band). Another band of marine epoxy was applied to an area of healthy coral ca. two to five centimeters beyond the edge of the primary band as a “firebreak” or a second attempt to block disease progression if the primary band failed to halt disease progression (secondary band) (Fig. 2).

We conducted a preliminary trial using this double—band method for a tissue loss disease elsewhere and found that treatment was more effective if the epoxy was mixed with chlorine powder. There is the possibility that the chlorine can diffuse out of the marine epoxy and affect adjacent coral tissue. However, there was little mortality of coral tissue beyond the 2^nd^ treatment bands suggesting, chlorine-impregnated epoxy caused minimal damage to the colony. This was confirmed after several months, whereupon corals were observed re-growing back over the epoxy, that itself, was colonized by filamentous and crustose coralline algae (Fig. 2) suggesting minimal longer-term toxicity.

All colonies (control and experimental) were photographed before and after marking or treatment and re-photographed two months later. All of the tagged colonies were flat, encrusting or plating colonies, thereby allowing rate of tissue loss per day to be measured digitally (Image J software, v1.46 NIH). The perimeter of the healthy tissue was digitally outlined and the encircled area calculated in cm^2^. Digital measurements were conducted in triplicate for each photograph and the averages were used for analysis.

### Identification of bacteria indicative of BBD

To determine if the dominant microbial constituents in BBD were present in the lesions characteristic of *Montipora* banded tissue loss, microscopy, culture, and PCR were used to identify representative filamentous cyanobacteria, sulfide-oxidizing bacteria, and sulfate-reducing bacteria from lesion material.

#### Isolation and growth of cyanobacteria.

Filamentous cyanobacteria identified under 10X magnification were removed from disease samples using sterile forceps, rinsed thrice in autoclaved filtered seawater (FSW), plated onto the center of a plate of solid ASW:BG-11 medium [27], and incubated at 28°C under 2.0% CO_2_ and 30 to 40 μE/m^2^ /sec cool white fluorescent lighting. A subpopulation of filaments that had migrated to the periphery of the plate was transferred to fresh medium weekly. Bacteria were passaged five times before identification. Standard microscopy of filamentous bacteria was conducted [28].

#### Isolation and growth of sulfide-oxidizing bacteria.

White filamentous bacteria containing large inclusion bodies, grossly similar to *Beggiatoa* spp., were removed from disease samples using a micropipettor and placed at the surface of J3 media deeps [29] within sterile polyethylene conicals, and buffered to pH 8.4 with Tris base, a medium designed to enrich sulfide-oxidizing bacteria. Inoculated J3 deeps were incubated at 28°C. Bacteria that were able to migrate into the J3 deep would indicate a motile bacterium capable of living in a micro-oxic, sulfide-rich environment, such as *Beggiatoa* spp.. Bacteria that migrated into the deep were re-isolated with a syringe penetrating through the side of the conical and re-inoculated onto the surface of fresh medium. Bacteria were passaged seven times before identification.

#### Molecular identification of bacteria.

To identify marine cyanobacteria, filaments either removed directly from sampled lesions or strains cultured on ASW:BG-11, were boiled in sterile Milli-Q water for five minutes, centrifuged at 5000 x g for two minutes, and the supernatant was used as template DNA for PCR amplification of the16S rRNA genes using the cyanobacteria-specific primers CYA106F and CYA781R(a) [30]. PCR products from cyanobacterial filaments were sequenced directly using primer CYA106F.

To identify filamentous sulfide-oxidizing bacteria that were cultured as described above or taken directly from lesions, filaments were boiled in Milli-Q water for five minutes, centrifuged at 5000 x g for two minutes, and the supernatant was used as template DNA for PCR amplification of the 16S rRNA gene using universal bacterial primers 8F and 1513R [31].

The *dsrA* gene encodes subunit A of dissimilatory sulfite reductase and has been used to indicate the presence of sulfate-reducing bacteria in environmental samples, including material from BBD [23, 32–34]. Total DNA was extracted from coral lesion material using the PowerSoil DNA Extraction kit (MoBio) as per the manufacturer’s instructions. DNA from lesions was used as a template for PCR amplification of an approximately 200 bp region of the *dsrA* gene using primers DSR1-F and DSR-R [32], which target a subset of *dsrA* gene sequences previously identified from sulfate-reducing bacteria [35] and from *Desulfovibrionacae* of BBD microbial mats [23, 32–34].

PCR products derived from the 16S rRNA gene of filamentous sulfide-oxidizing bacteria or *dsrA* genes of sulfur-reducing bacteria were cloned into the *Sma*I site of pBlueScript SK+ as previously described and sequenced using the primers M13F and M13R [36].

### Data analyses

The rate of tissue loss of infected colonies was compared between treated and control colonies of *M*. *capitata*. The data were not normally distributed so a non-parametric Mann-Whitney two-group test was used to assess treatment effectiveness. The proportion of the colony that was healthy tissue at time zero (T0) and after two months (T1) was compared for treated and control corals.

Phylogenetic analysis of DNA sequences was conducted as previously described [37]. Briefly, sequences were aligned using BioEdit [38] and a maximum likelihood tree was constructed using the generalized time-reversible (GTR) algorithm [39] and 1000 bootstrap replicates were performed using MEGA5 [40]. All positions containing gaps and missing data were eliminated using the Maximum Likelihood method [41]. All *dsrA* sequences were checked manually for chimeras using alignment in MEGA5 and BLAST before analysis. All 16S rRNA gene sequences and *dsrA* sequences identified in this study were deposited in the GenBank database under accession numbers KM924158–60, KJ914890–91, KM258122–127, KM924161–163, 165.

## Results

### Rapid surveys

At Anini site one, 133 montiporid colonies were examined and 8 showed signs of the disease (prevalence = 6.0%). At Anini site two, 170 montiporids were examined and 13 were found infected (prevalence = 7.6%).

### Rate of tissue loss in control and treated colonies

*M*. *capitata* colonies treated with a double band of marine epoxy lost significantly less tissue as compared to the untreated control colonies.(Mann-Whitney test, W = 97.0, p = 0.003). Average tissue loss was 1.1 (SE± 0.4) cm^2^/day for treated colonies compared to untreated colonies averaging 5.7 (SE±1.1) cm^2^ of tissue loss per day.

Colony sizes were similar between groups with control colonies having an average total surface area (healthy and dead) of 692.7 cm^2^ (range 148.6–2881.1 cm^2^) and treated colonies had an average of 703.4 cm^2^ (range 72–1556.2 cm^2^). The average proportion of dead area on colonies at the beginning of the study was also similar in both control and treatment groups (9.7 ± 2.5% and 10.4 ± 1.2% respectively, Mann-Whitney, W = 60.0, p = 0.431). After two months, however, untreated control colonies exhibited significantly more mortality (avg. reduction in area of healthy tissue = 69.7 ± 10.6%) compared to treated colonies (avg. = 23.4 ± 7.1%) (Mann-Whitney test, W = 94.0, p = 0.007) (Fig. 2) and two control colonies experienced 100% mortality (case fatality rate = 25%).

For treated colonies, one colony had no tissue loss beyond the primary band and four additional colonies showed no tissue loss beyond the secondary band during the observation period. The remaining three out of eight treatment colonies experienced minimal tissue loss past the secondary band (avg. 0.52 cm^2^/day).

### Identification of the pathogens indicative of BBD

#### Cyanobacteria associated with disease lesions.

**Fig 3:**
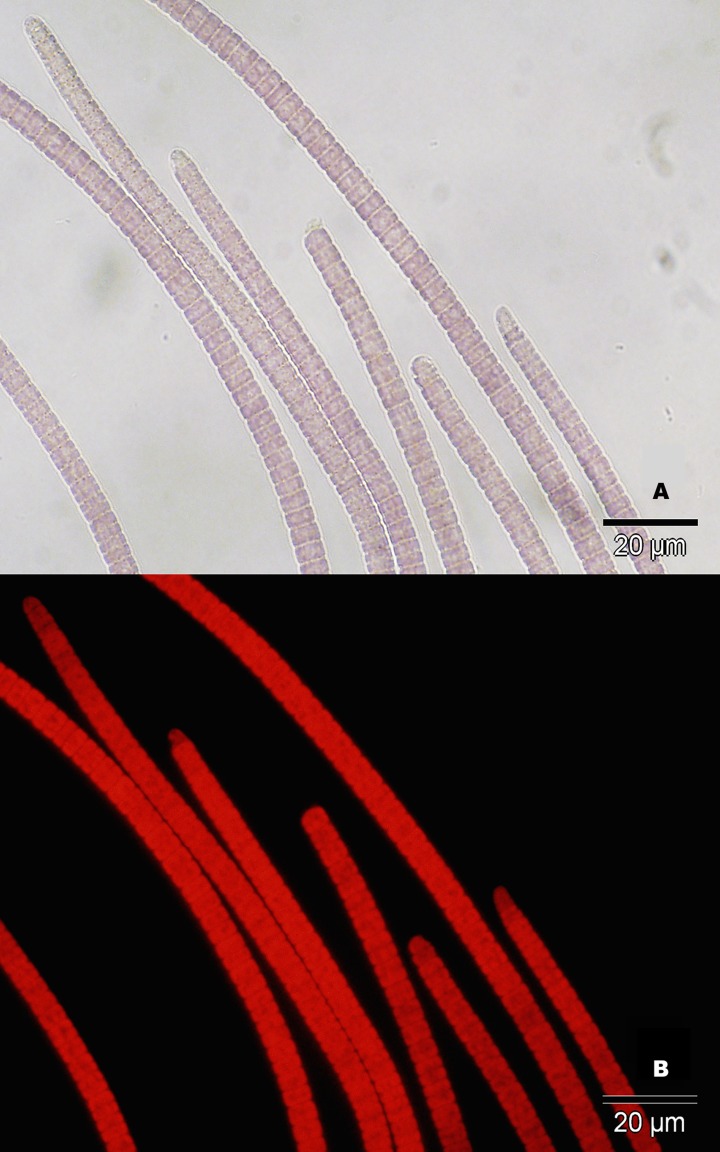
Microscopy images of cyanobacteria isolated from disease lesions. A) Cyanobacteria under light microscope. B) Cyanobacteria under fluorescence microscope. Red autofluorescence is indicative of photosynthetic pigments. Images were taken at 60x magnification. Black bar represents 20 μm.

**Fig 4:**
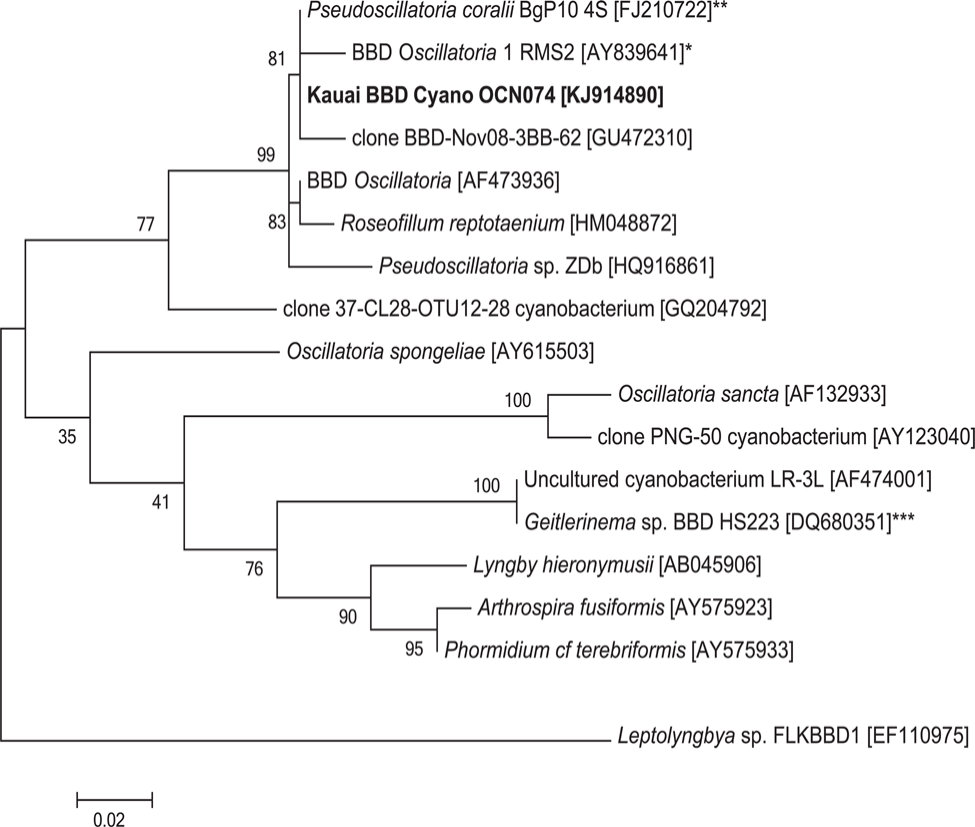
Phylogenetic relationship of cyanobacterium OCN074, isolated from disease lesions from Kaua‘i, to other cyanobacteria. All 16S rRNA gene sequences from the three Kaua‘i BBD cyanobacterial isolates were identical. A phylogenetic tree was generated using the Maximum Likelihood method. The ‘*’ indicates cyanobacteria associated with BBD of Palau (*[42]) the Red Sea (**[41]), and the Florida Keys (***[43]). The tree with the highest log likelihood is shown, and 1000 bootstrap replicates were used. NCBI accession numbers are in brackets and bootstrap values are indicated at branch nodes. Scale bar represents two substitutions per 100 nucleotide positions.

The visually dominant filamentous microorganism from the disease lesions exhibited a gross morphology and typical autoflorescence characteristic of cyanobacteria (Fig. 3). 16S rRNA gene sequences from filaments taken directly from disease lesions and two cultured strains, Kaua‘i isolates OCN073 and OCN074, were identical to each other (KJ914890). The sequences from Kaua‘i cyanobacterial isolates were 99% identical to that of *Pseudoscillatoria coralii* strain BgP10_4S, a cyanobacterium associated with BBD from the Red Sea [42], and *Oscillatoria* sp. RMS2, the cyanobacterium associated with BBD from Palau [43]. The Kaua‘i isolates clustered with cyanobacteria associated with BBD from regions in the Indo-Pacific but did not cluster with the *Geitlerinema* sp. isolate (previously described as *Phormidium corallyticum*), the original BBD cyanobacterium isolated from the Florida Keys [44] (Fig. 4).

#### *Beggiatoa* associated with disease lesions.

**Fig 5:**
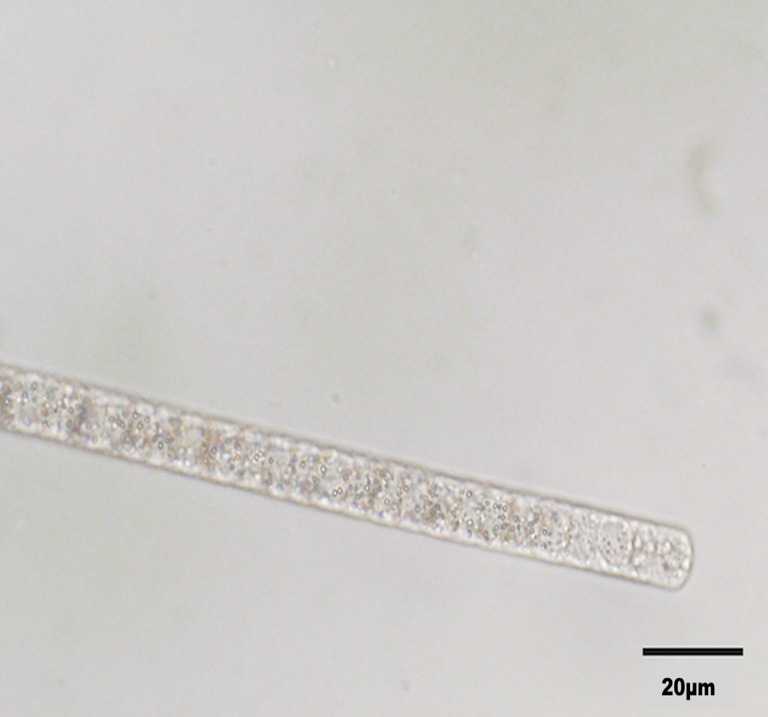
Brightfield image of Beggiatoa sp. filaments removed from disease lesions. Image taken at 60x magnification. Black bar represents 20 μm.

**Fig 6:**
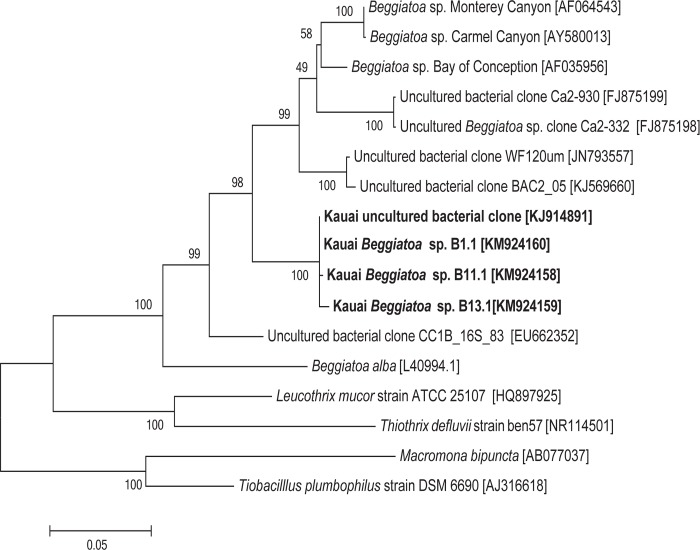
Phylogenetic relationship of *Beggiatoa* spp. isolated from disease lesions from Kaua‘i to other *Beggiatoa*. A phylogenetic tree was generated using the Maximum Likelihood method with sequences from 14 other *Beggiatoa* and closely related sulfur-oxidizing strains including representative type strains from the Thiotrichaceae family. No 16S rRNA gene sequences from *Beggiatoa* found in BBD from other regions were available for comparison. The tree with the highest log likelihood is shown, and 1000 bootstrap replicates were used. NCBI accession numbers are in brackets, and bootstrap values are indicated at branch nodes. Scale bar represents five substitutions per 100 nucleotide positions.

White filamentous microorganisms that contained apparent sulfur granules and exhibited gliding motility, consistent with *Beggiatoa*, were observed from samples of coral lesions (Fig. 5). Inoculation of J3 medium, which is rich in hydrogen sulfide, resulted in the migration of filaments two to three centimeters down into the deep, consistent with the motile behavior of sulfide-oxidizing *Beggiatoa* [28]. Sequences of 16S rRNA genes from filaments cultured on J3 deeps (KM924160, KM924158, KM924159) or taken directly from lesion material (KJ914891) clustered together (99 to 100% identical) and with other *Beggiatoa* species (Fig. 6). Survival and behavior in hydrogen sulfide rich medium, the presence of large granules, gliding motility, and 16S rRNA gene sequence are all consistent with these isolates belonging to the genus *Beggiatoa*.

#### Sulfate-reducing bacteria associated with disease lesions.

**Fig 7:**
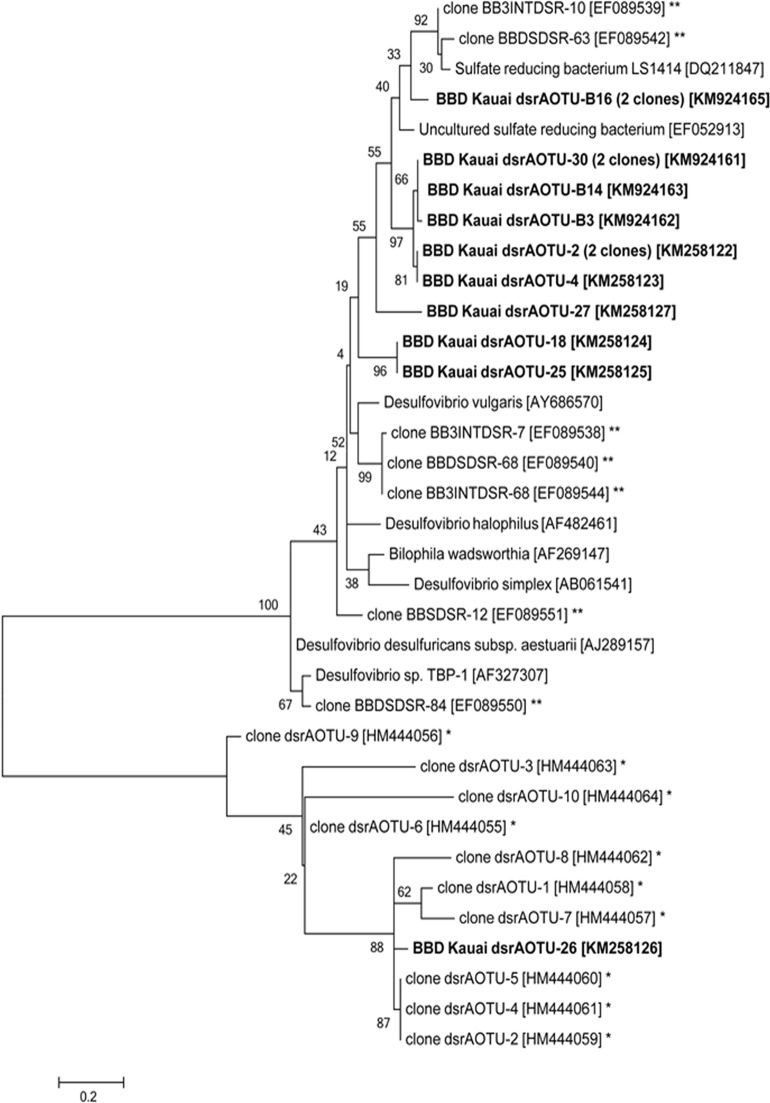
Phylogenetic relationship of sulfate-reducing bacteria from disease lesions from Kaua‘i to other sulfate-reducing bacteria. A phylogenetic tree was generated using the Maximum Likelihood method with a reference sequence library built from 25 known *dsrA* gene sequences used as a proxy for the presence of sulfate-reducing bacteria [23, 33]. The ‘*’ indicates gene sequences from BBD samples previously published from the Great Barrier Reef (* [33]), and the Red Sea (** [23]). The tree with the highest log likelihood is shown, and 1000 bootstrap replicates were used. NCBI accession numbers are in brackets, and bootstrap values are indicated at branch nodes. Scale bar represents two substitutions per 100 nucleotide positions.

A diverse group of sulfate-reducing bacteria were present in the Kaua‘i coral lesions. The *dsrA* gene was used as a proxy for the presence of sulfate-reducing bacteria [35, 45] and partial *dsrA* sequences were recovered by PCR using *dsrA*-specific primers from lesion material. Thirteen sequences were determined and compared to those from other BBD samples and known sulfate-reducing bacteria (Fig. 7). Two general groups of sulfate-reducing bacteria were identified after phylogenetic analysis. Twelve sequences from the Kaua‘i BBD lesion (KM258124, KM258125, KM258127, KM258122 (2 clones), KM258123, KM924162, KM924163, KM924161 (2 clones), KM924165 (2 clones)) clustered with sequences retrieved from the Red Sea BBD lesion material [23] and sequences from other *Desulfovibrio* strains. One Kaua‘i *dsrA* sequence (KM258126) clustered with *dsrA* sequences retrieved from the Great Barrier Reef BBD lesion material [34].

## Discussion

This study establishes the presence of black band disease (BBD) on montiporids in the Hawaiian archipelago. The three major microbial components indicative of BBD (filamentous cyanobacteria, sulfide-oxidizing bacteria and sulfate-reducing bacteria) were identified in disease lesions from corals on Kaua‘i. A phototrophic, filamentous, motile cyanobacterium cultured from BBD lesions in Kaua‘i was genetically similar to both *Pseudoscillatoria* and *Oscillatoria*, cyanobacteria found in BBD reported from other areas of the Indo-Pacific [43]. Motile, white filamentous bacteria from Kaua‘i BBD lesions were morphologically similar to other strains of *Beggiatoa* [46], and corresponding 16S rRNA gene sequences were identified as *Beggiatoa*. Prior studies on BBD have exclusively used the distinct morphology of *Beggiatoa* to identify this bacterium [24, 25]. Our study is the first to use morphology, behavior and molecular analysis for identification. Lastly, a range of *dsrA* gene sequences were amplified from the Kaua‘i BBD lesions, indicative of a diverse group of bacteria that can reduce sulfate. No DNA sequences were available for *Beggiatoa* found in BBD from other regions for our comparison. However, consistent with other studies, we found that although BBD is found on reefs worldwide, the specific bacterial species associated with this polymicrobial disease that have been genetically identified (cyanobacteria and sulfate-reducing bacteria) varies across regions [23, 47, 48]. It has been suggested that the specific microbial communities found in BBD may be primarily derived from the local reef environment [48], which could explain the regional differences in species found in BBD bacterial communities. More phylogenetic studies on *Beggiatoa* are needed to further investigate this hypothesis.

Our study extends the known range of BBD within the Indo-Pacific, which has previously been documented in Palau [43], Great Barrier Reef (GBR) [4], Guam [49], American Samoa [6], Indonesia [50], Philippines [51], Japan [52], Arabian Gulf [53], and the Red Sea [54]. Prior to this study, no occurrences of BBD were reported in Hawai‘i despite extensive disease surveys [5, 12, 13].

An Eyes of the Reef (EOR) member, who had been trained in disease identification, first reported the disease outbreak and the outbreak status of the disease was confirmed by rapid surveys as outlined in Hawai‘i’s rapid response contingency plan (RRCP) [18]. At the alleged outbreak site (Anini) on Kaua‘i’s north shore, disease prevalence was found to be 6 to 7%, which is higher than baseline levels of the disease, previously referred to as *Montipora* banded tissue loss, recorded in nearby Hanalei Bay, Kaua‘i (prevalence <1%) [19]. Such high levels of disease compared to baseline levels constitute an epizootic. Since the disease was found to be at outbreak levels, a multi-agency research response was initiated. Having the ability to identify and respond quickly to disease outbreaks highlights the value of programs such as EOR and Hawai‘i’s RRCP. The need for standardized disease response protocols has been recognized elsewhere and resulted in the recent creation of multiple resources for biologists [55–57]. The importance of having prior knowledge of baseline disease levels cannot be overemphasized; it gives scientists and managers the capacity to identify and respond to changes in disease levels through time. Coral reefs are declining precipitously [58, 59] and, in response to continued problems associated with anthropogenic overuse and global climate change, coral disease outbreaks are predicted to increase over time [60]. Numerous regions throughout the Indo-Pacific lack baseline coral disease surveys, which highlight the critical need for agencies, managers and scientists to work together to fill in these gaps in knowledge.

In other regions, BBD usually occurs at a low prevalence (<1%) but is considered a disease of concern because it is a chronic disease on reefs, often persisting for years and contributing to the long-term decline of susceptible coral species [61–63]. In Hawai‘i, *Montipora* banded tissue loss, which is now known to be BBD, was first found in Hanalei Bay on Kaua‘i in 2004 [19], and found in subsequent surveys in 2007 and 2009 (S1 Table). Hence, we know the disease has been affecting montiporids on Kaua‘i for at least 10 years. We also found that BBD can cause significant colony mortality on *Montipora capitata* with colonies losing an average of 68.7% of their live tissue within two months. In other regions, BBD prevalence and virulence is seasonal with higher prevalence and rate of tissue loss found during the warmer months [33, 61]. Our study occurred from September to November; months that coincide with warmer water temperatures in Hawai‘i (http://www.nodc.noaa.gov/dsdt/cwtg/hawaii.html), which may partly explain the high rate of tissue loss observed. BBD was also found to be more virulent than other tissue loss diseases in Hawai‘i. For example, *Montipora* white syndrome (MWS) is a tissue loss disease affecting *M*. *capitata* colonies in Kāne‘ohe Bay, Oahu. MWS-affected colonies lost an average of 3% of the colony per month [16], roughly one tenth the rate observed with colonies affected by BBD on Kaua‘i.

Coral disease treatment has now been successfully used for a number of coral diseases [8, 64, 65] and we found that disease treatment with a double band of marine epoxy mixed with chlorine powder significantly reduced colony mortality from BBD. In five out of eight colonies, treatment was sufficient to stop disease progression (63% effective) and in the remaining three colonies, in which the disease front crossed both treatment bands, tissue loss was significantly reduced compared to controls. Hudson [64] also successfully treated black band disease (70% effective) by removing the pathogen by suction and covering the affected area with modeling clay. In Australia, mechanical removal of the advancing disease margin for *Turbinaria* colonies affected by a tissue loss disease (“white syndrome”) was successful at halting the disease in 80% of the colonies [8]. Removal of growth anomalies on branching acroporids was an effective treatment with 90% of colonies remaining disease free for nine months post-treatment [65]. Although disease treatments are a temporary fix that address the signs, not the underlying causes of disease, they may be useful for containing disease outbreaks, if caught early, or for reducing morbidity and mortality from outbreaks. As such, the different disease treatments being developed should be considered by resource managers as they continue to address the growing threats from coral disease events.

BBD is known to affect multiple host genera [3] but on Kaua‘i it was only observed on three species of *Montipora* (*M*. *capitata*, *M*. *flabellata*, *M*. *patula*) even though *Porites*, known to be BBD susceptible in other regions [3], is also commonly found on the reefs surveyed. While further surveys on Kaua‘i may reveal other infected genera, it initially appears that in Hawai‘i, montiporids are the primary coral genera affected by BBD. Similarly, acroporids consistently have the highest BBD levels on the GBR, although numerous genera are susceptible [4, 63].

Interestingly, *Montipora* spp. are abundant throughout the reefs of Hawai‘i [66, 67] yet BBD has only been found on Kaua‘i despite extensive baseline disease surveys throughout the Hawaiian archipelago [5, 12, 13]. What is different about the north shore of Kaua‘i that might be allowing BBD to persist and reach outbreak levels? Coastal coral reefs are increasingly exposed to excess nutrients, sediments, and pollutants discharged from land. Coastal development, agriculture, and overgrazing have all contributed to increased terrestrial runoff and numerous studies have shown that sedimentation, turbidity, and nutrient enrichment can degrade local coral reefs [68]. The north shore of Kaua‘i has been plagued by chronic, impaired coastal waters having high levels of sedimentation [69], excess nutrient loading, and bacterial contamination [70, 71]. At the sites we surveyed for coral disease, there were obvious signs of excessive sedimentation and sediment damage on corals. Increases in coral diseases have also been found associated with reduced water quality from terrestrial run-off. In Australia, a 10-fold greater mean abundance of disease was found on reefs during the rainy summer months and it was concluded that rainfall and associated runoff were facilitating disease outbreaks [72]. Increased prevalence of BBD in the field has been associated with sewage effluent [73] and laboratory tests showed that the rate of tissue loss from BBD was increased with nutrient enrichment [74]. When experimental *in situ* nutrient enrichment of reefs in the Caribbean were conducted, corals exposed to chronic nutrient stress suffered a 3.5-fold increase in bleaching frequency and a two-fold increase in prevalence and severity of disease, compared to corals in control plots [75]. Additionally, nine months after removal of the nutrients, there were no differences in bleaching or disease levels in experimental versus control plots. This suggests that improvement in water quality may be an effective way to mitigate some coral diseases and improve overall coral reef integrity. It is likely that the chronically degraded water quality found on Kaua‘i’s north shore is contributing to the spread and severity of coral disease. Further studies are currently underway to examine this hypothesis.

## Supporting Information

S1 Table Disease surveys conducted within Hanalei Bay, Kauai in 2007 and 2009. (DOCX)
