# Supplementary material for: First Record of Black Band Disease in the Hawaiian Archipelago: Response, Outbreak Status, Virulence, and a Method of Treatment
Source: PLoS One. 2015 Mar 16;10(3):e0120853. doi: 10.1371/journal.pone.0120853 (PMC4361573; doi:10.1371/journal.pone.0120853)
Supplement: S1 Table — (DOCX) [file pone.0120853.s001.docx]

**S1 Table** . **Disease surveys conducted within Hanalei Bay, Kauai in 2007 and 2009.** Two 25 meter belt transects were surveyed per site. Coral cover determined by point-intercept method at 50 cm intervals. BBD=black band disease.

| **year** | **# sites surveyed** | **total reef area surveyed (m^2^)** | **Avg. *Montipora* cover (%)** | **Total # BBD cases** | **# BBD/m^2^ *Montipora*** | **Est. BBD prevalence (%)** |
| --- | --- | --- | --- | --- | --- | --- |
| 2007 | 3 | 900 | 11.1 | 11 | 0.11 | 0.78 |
| 2009 | 2 | 600 | 23.2 | 11 | 0.08 | 0.53 |
